# Eastern Equine Encephalitis Virus in Mosquitoes and Their Role as Bridge Vectors

**DOI:** 10.3201/eid1612.100640

**Published:** 2010-12

**Authors:** Philip M. Armstrong, Theodore G. Andreadis

**Affiliations:** Author affiliation: The Connecticut Agricultural Experiment Station, New Haven, Connecticut, USA

**Keywords:** Vector-borne infections, eastern equine encephalitis virus, viruses, mosquitoes, Connecticut, vectors, research

## Abstract

Eastern equine encephalitis virus (EEEV) is maintained in an enzootic cycle involving *Culiseta melanura* mosquitoes and avian hosts. Other mosquito species that feed opportunistically on mammals have been incriminated as bridge vectors to humans and horses. To evaluate the capacity of these mosquitoes to acquire, replicate, and potentially transmit EEEV, we estimated the infection prevalence and virus titers in mosquitoes collected in Connecticut, USA, by cell culture, plaque titration, and quantitative reverse transcription–PCR. *Cs. melanura* mosquitoes were the predominant source of EEEV (83 [68%] of 122 virus isolations) and the only species to support consistently high virus titers required for efficient transmission. Our findings suggest that *Cs. melanura* mosquitoes are primary enzootic and epidemic vectors of EEEV in this region, which may explain the relative paucity of human cases. This study emphasizes the need for evaluating virus titers from field-collected mosquitoes to help assess their role as vectors.

## Abstract 2

TOC summary: Virus titers are useful for assessing which mosquito species may transmit virus.

During the past 6 years, eastern equine encephalitis virus (EEEV; family *Togaviridae*, genus *Alphavirus*) has reemerged in the northeastern United States and resulted in 26 human cases of infection and 9 deaths (ArboNET; Centers for Disease Control and Prevention, Atlanta, GA, USA). Virus transmission has intensified throughout this region, spread to locales where it had not been previously detected, and extended north into New Hampshire and Maine, USA, and Nova Scotia, Canada (*1*). Disease outbreaks caused by EEEV occur at irregular intervals when underlying ecologic conditions favor virus amplification and overflow into human and equine populations.

EEEV is perpetuated in an enzootic cycle involving ornithophilic mosquitoes (primarily *Culiseta melanura*) and passerine birds in freshwater swamps (*2**,**3*). Human and equine cases occur infrequently despite relatively high rates of EEEV infection in *Cs. melanura* during virus amplification (ArboNET). Other mosquito species such as *Aedes vexans*, *Coquillettidia perturbans*, *Ochlerotatus canadensis*, and *Oc*. *sollicitans* have been implicated as epidemic/epizootic bridge vectors from viremic birds to horses and humans (*4**–**6*). These species are competent vectors of EEEV (*7**–**9*) and may acquire virus infection during disease outbreaks by feeding occasionally on birds but prefer mammalian hosts (*10**–**14*). Although *Cs. melanura* mosquitoes feed infrequently on mammals (*10**,**12**,**15*), their ability to serve as a bridge vector may be offset by a much higher prevalence of EEEV infection in this species.

One criterion used for incriminating enzootic and bridge vectors is based on the frequency of virus detection from each candidate species (*16*). Typically, mosquitoes are collected from disease-endemic sites, sorted into pools by trap location and species, and screened for virus by cell culture or molecular methods. This procedure provides critical information on the identity, spatial and temporal distribution, and proportion of virus-infected mosquitoes and forms the basis for many arbovirus surveillance programs. Nevertheless, virus titers may vary considerably within infected mosquitoes (*17*) and reflect the duration of the extrinsic incubation period and the ability of these mosquitoes to support virus replication, which is a necessary precondition for mosquitoes becoming infectious (*18*). The virus must undergo several rounds of replication in the mosquito midgut and salivary glands before being biologically transmitted to the vertebrate host when the mosquito salivates during blood feeding.

In Connecticut in 2009, EEEV activity increased substantially, and we isolated numerous viruses from *Cs. melanura* mosquitoes and potential bridge vectors. To evaluate the capacity of these mosquitoes to replicate and potentially transmit virus, we estimated EEEV titers from virus-positive mosquito pools with the expectation that the most efficient vectors will support consistently high virus titers.

## Materials and Methods

### Mosquito Collections

Mosquitoes were collected at 91 trapping locations statewide as a part of the Connecticut Mosquito and Arbovirus Surveillance Program during June–October 2009. Each trapping site was sampled on average weekly and at least every 10 days by means of CO_2_-baited Centers for Disease Control light traps and gravid traps that were operated overnight. Adult mosquitoes were transported back to the laboratory alive and sorted on chill tables by trap location and according to species by using taxonomic keys (*19*). Mosquitoes containing visible blood in the abdomen were removed and not included in this study. Mosquitoes were combined into pools of <50 individuals, placed in microcentrifuge tubes containing a copper BB, and stored at –70°C until virus testing.

### Virus Isolation and Identification

Mosquitoes were homogenized in 1.0–1.5 mL of phosphate-buffered saline supplemented with 30% heat-inactivated rabbit serum, 0.5% gelatin, and antibacterial and antifungal drugs by using a vibration mill set for 4 min at 25 cycles/s. Homogenates were centrifuged at 4°C for 7 min at 520 × *g*, and 100 µL of the supernatant was placed on confluent Vero cells growing in 25-cm^2^ flasks containing minimal essential media, 5% fetal bovine serum, and antibacterial and antifungal drugs. Cell cultures were maintained at 37°C in an atmosphere of 5% CO_2_ and monitored daily for cytopathic effect during days 3–7 postinfection. Infected cell supernatants were harvested and stored at –80°C.

Mosquito pools that yielded infectious virus in cell culture were directly tested for EEEV by quantitative reverse transcription–PCR (qRT-PCR). RNA was extracted from mosquito pool homogenates by using spin columns and reagents in a viral RNA kit (QIAGEN, Valencia, CA, USA). A total of 2.5 µL of this preparation was added to a 25-µL qRT-PCR by using the TaqMan One-Step RT-PCR Kit (Applied Biosystems, Foster City, CA, USA) and primers/probe (9391/9459c/9414probe) (*20*). Amplification was performed as follows: 1 cycle at 50°C for 20 min and 95°C for 10 min and 50 cycles at 95°C for 15 s and 60°C for 1 min. Positive results were based on cycle threshold (C_t_) values when the change in fluorescence increased above the baseline threshold value calculated by using IQ5 Optical System Software (Bio-Rad, Hercules, CA, USA). Samples that failed to amplify or yielded C_t_ values >30 were reconfirmed by reisolation of EEEV in cell culture. Supernatants from virus cultures were also tested for EEEV by qRT-PCR or by conventional RT-PCR and by sequencing a portion of the nonstructural protein gene of EEEV as described (*21*).

### Plaque Titration

Plaque titrations were performed on confluent Vero cell cultures growing in 12-well plates. Ten-fold dilutions of mosquito pool homogenates were placed in triplicate onto cell monolayers and absorbed for 1 h at 37°C in an atmosphere of 5% CO_2_. Cells were overlaid with 1% methylcellulose in minimal essential medium, 5% fetal bovine serum, and antimicrobial and antifungal drugs and returned to the incubator. After 3 days, cells were fixed overnight in 7.4% formaldehyde and stained with 1% crystal violet so plaques could be visualized.

### Statistical Analyses

The Pooled Infection Rate add-in for Excel (Microsoft, Redmond, WA, USA) was used to calculate virus infection rates (per 1,000 mosquitoes) and 95% confidence intervals (CIs) on the basis of maximum likelihood estimation (MLE) (*22*). All other statistical tests were performed by using Instat version 3.06 (GraphPad Software, San Diego, CA, USA). The Mann-Whitney U test was used for comparing median virus titers in pools for *Cs. melanura* mosquitoes and 6 other species from which a minimum of 3 positive pools were obtained (*Ae. cinereus*, *Anopheles punctipennis*, *Culex salinarius*, *Oc. canadensis*, and *Uranotaenia sapphirina* mosquitoes). The relationship between C_t_ values and log_10_ virus titers were analyzed by regression analysis. Statistical significance was assigned at p<0.05 or by nonoverlapping 95% confidence intervals.

## Results

In 2009, a total of 291,641 mosquitoes (35 species) were collected and processed as 16,909 pools for virus isolation. EEEV was isolated from 122 mosquito pools, which represented 14 species and 7 genera (Table), obtained during August 17–October 27 in 25 of 91 trapping locations. *Cs. melanura* mosquitoes yielded the greatest number of EEEV isolations (n = 83) and was followed by *Oc*. *canadensis* (n = 10) and *Ae*. *cinereus* (n = 6) mosquitoes. Relatively few (<4) or no EEEV isolates were obtained from the remaining mosquito species. EEEV infection rates were higher in *Cs. melanura* mosquitoes (MLE 3.44, 95% CI 2.76–4.24) than in all other mosquito species tested except *An*. *quadrimaculatus* (MLE 3.27, 95% CI 0.59–10.55) and *Ur*. *sapphirina* (MLE 1.35, 95% CI 0.44–3.23) mosquitoes.

**Table:** Eastern equine encephalitis virus isolated and virus titers from mosquitoes obtained in Connecticut, USA, 2009*

Mosquito species	No. mosquitoes collected	No. virus isolates	Infection rate/1,000 mosquitoes, MLE (95% CI)	Mean C_t_ by qRT-PCR	Mean titer log_10_ PFU/ mosquito pool	% Mosquito pools >3.0 log_10_ PFU/ mosquito pool
*Aedes cinereus*	15,294	6	0.4 (0.2–0.8)	34.0	2.92	16.7
*Ae. vexans*	26,462	2	0.1 (0–0.2)	33.0	1.15	0
*Anopheles punctipennis*	5,573	4	0.7 (0.2–1.7)	34.6	1.69	0
*An. quadrimaculatus*	607	2	3.3 (0.6–10.6)	35.9	1.67	0
*An. walkeri*	2,381	2	0.8 (0.2–2.7)	35.1	1.43	0
*Culex restuans*	14,609	1	0.1 (0–0.3)	35.2	0.85	0
*Cx. salinarius*	12,605	3	0.2 (0.1–0.6)	32.1	1.31	0
*Culiseta melanura*	25,595	83	3.4 (2.8–4.2)	22.3	6.55	88.0
*Ochlerotatus canadensis*	40,543	10	0.2 (0.1–0.4)	32.9	2.82	10.0
*Oc. cantator*	4,457	1	0.2 (0–1.1)	31.8	1.54	0
*Oc. triseriatus*	3,000	1	0.3 (0–1.6)	>50	1.60	0
*Oc. trivittatus*	23,340	2	0.1 (0–0.3)	38.8	<0.8	0
*Psorophora ferox*	13,677	1	0.1 (0–0.35)	34.0	<0.8	0
*Uranotaenia sapphirina*	2,954	4	1.4 (0.4–3.2)	36.5	1.00	0
Remaining species†	100,544	0	–	–	–	–

*MLE, maximum-likelihood estimation; CI, confidence interval; C_t_, cycle threshold; qRT-PCR, quantitative reverse transcription–PCR; –, not applicable. Mean C_t_ and PFU values were calculated for mosquito pools positive by qRT-PCR and plaque titration. †n = 21.

Mosquito pools that yielded EEEV in cell culture were directly tested by qRT-PCR. A total of 108 mosquito pools had positive C_t_ values (<37) on the basis of criteria established by Lambert et al. (*20*), 7 mosquito pools were equivocal (C_t_ 37.6–39.0), and 7 isolates failed to amplify after 50 amplification cycles. All mosquito pools that were negative or had C_t_ values >30 by qRT-PCR were retested in Vero cell culture and confirmed by reisolation of EEEV. Mean C_t_ values were lowest for *Cs. melanura* mosquitoes (C_t_ 22.3) and exceeded 30 for all other mosquito species (Table), which suggested species-specific differences in virus titer.

Concentration of infectious virus was estimated from positive mosquito pools by plaque titration in Vero cell culture. *Cs. melanura* mosquitoes had significantly higher virus titers (mean 6.55 log_10_ PFU/mosquito pool) than all other mosquito species for which statistical comparisons were possible (p<0.01 by Mann-Whitney U test). *Ae*. *cinereus* and *Oc*. *canadensis* had the next highest virus titers (2.92 log_10_ PFU/mosquito pool and 2.82 log_10_ PFU/mosquito pool, respectively), and mean titers ranged from <0.8 log_10_ PFU/mosquito pool to 1.69 log_10_ PFU/mosquito pool in the remaining species. The percentage of *Cs. melanura* mosquito pools with high virus titers (>3.0 log_10_ PFU/mosquito pool) was 88% compared with 0%–16.7% for the 13 other mosquito species (Table).

The relationship between C_t_ values and PFU estimated from EEEV-infected mosquito pools is shown in the Figure. A strong negative correlation was found between C_t_ values and PFU/mL in positive mosquito pools by regression analysis (slope –3.1, y-intercept 39; p>0.0001), which indicated a predictive relationship between these 2 measures of virus concentration.

**Figure:**
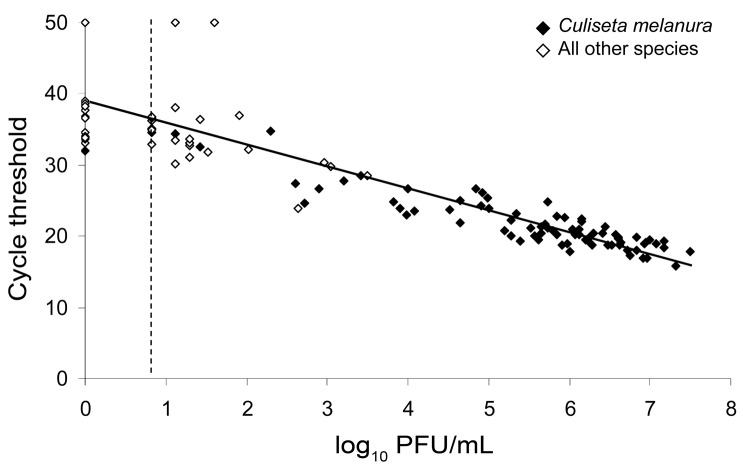
Relationship between cycle threshold value and PFU estimated from eastern equine encephalitis virus–infected mosquito pools, Connecticut, USA, 2009. Mosquito pools negative for virus by plaque titration were assigned a value of 0, and mosquito pools negative by quantitative reverse transcription–PCR were assigned a value of 50. Limit of detection by plaque titration (0.8 log_10_ PFU/mL) is indicated by the dashed vertical line.

## Discussion

Our analysis of EEEV-positive mosquito pools showed major differences in virus titer among different mosquito species obtained in Connecticut. *Cs. melanura* was the only species in which titers developed that were associated with EEEV transmission, estimated to be 4–7 logs of virus in virus-transmitting mosquitoes in previous studies (*23**–**25*). This finding suggests that EEEV is transmitted primarily by *Cs. melanura* mosquitoes in this region of the northeastern United States, despite repeated virus isolations from other mosquito species.

Infrequent human and horse infections by EEEV may arise when *Cs*. *melanura* mosquitoes occasionally feed on mammals rather than by participation of another epidemic/epizootic bridge vector. Prior studies identified mammalian-derived blood meals in 1%–10% of *Cs*. *melanura* mosquitoes obtained in Massachusetts, Connecticut, and New York (*10**,**12**,**15*) and provided a direct conduit for virus transmission by this species to horses and humans. These analyses, in conjunction with observations on vector longevity (*26*), vector competence (*8**,**25**,**27*), and prevalence of EEEV infection in *Cs*. *melanura* mosquitoes (ArboNET), suggest that this species could serve as an enzootic and epidemic/epizootic bridge vector of EEEV. We provide additional support for this hypothesis by estimating virus titers in field-collected mosquitoes, which enabled us to determine which infected mosquitoes could potentially transmit virus.

In this study and previous studies, most EEEV isolations have been from either *Cs*. *melanura* or *Cs*. *morsitans* mosquitoes, depending on the region. These 2 species comprised 46%–100% of all isolations from field-collected mosquitoes in published studies (*1**,**28**–**35*) and 62%–92% of all EEEV-positive mosquito pools reported to the Centers for Disease Control and Prevention through ArboNET during 2004–2009. The remaining virus isolations come from a diversity of species, some of which were implicated as bridge vectors largely on the basis of local abundance, temporal and spatial distribution in relationship to human cases, and virus isolation during epidemics.

*Ae. vexans* mosquitoes are often mentioned as a possible bridge vector of EEEV in the northeastern United States (*1**,**3**,**5**,**12**,**33*). Their distribution and late season abundance overlap with distribution of human cases, and they will feed opportunistically on avian and mammalian hosts. However, vector competence trials have ranked this species as an inefficient vector in the laboratory (*9*); it failed to transmit virus in 1 study (*8*). Our results suggest a negligible role for this species. EEEV was isolated only twice from *Ae. vexans* mosquitoes, and the 2 positive pools showed low virus titers, which reinforced previous findings. A study by Nasci and Mitchell (*17*) reported low EEEV titers (<3.0 log_10_ PFU/mL) in all *Ae. vexans* mosquito pools tested (n = 4).

*Oc*. *canadensis* mosquitoes were the second major source of EEEV after *Cs*. *melanura* mosquitoes and accounted for 10 (8%) of 122 virus isolations in this study. EEEV has been detected in this species throughout the northeastern United States, including New Jersey, Rhode Island, New York, Massachusetts, and New Hampshire (*33**,**34**,**36*) (ArboNET) and is a moderately competent vector in the laboratory (*8*). *Oc*. *canadensis* is the most frequently trapped mosquito in Connecticut and is found in a variety of habitats that include freshwater swamps in which EEEV is found. Adult populations peak in late June–early July but extend into fall (*19*), particularly if a second hatch occurs during periods of heavy rainfall. Host-seeking females feed mainly on mammals, including horses and humans and occasionally birds (*11**,**14*). This finding suggests that *Oc*. *canadensis* mosquitoes may be a bridge vector in Connecticut, but its relative contribution appears to be minor when virus titers in field-collected mosquito pools are considered. We detected high virus titers in only 1 pool of *Oc*. *canadensis* mosquitoes (3.2 log_10_ PFU/mL), which is consistent with observations in which 0 of 2 EEEV-positive pools of *Oc*. *canadensis* mosquitoes contained high titers of virus (>3.0 log_10_ PFU/mL) (*17*).

EEEV was also isolated from 6 *Ae. cinereus* mosquitoes, which represented the third most common source of virus. Of these pools, 1 contained high titers of virus (3.5 log_10_ PFU/mL). This species may serve as a potential bridge vector on the basis of certain ecologic and behavioral criteria. Host-seeking females are abundant during June–October in many habitats throughout Connecticut (*19*). This species feeds opportunistically on mammals and birds but prefers mammals (*11*). The ability of this species to transmit EEEV has not been evaluated in the laboratory. Therefore, its contribution as a vector requires further evaluation.

We did not isolate EEEV from *Oc*. *sollicitans* or *Cq. perturbans*, 2 mosquito species implicated as likely bridge vectors in other epidemiologic settings (*4**,**5**,**34**,**37*). The eastern salt marsh mosquito, *Oc*. *sollicitans*, is an aggressive biter of humans that may transmit virus in the mid-Atlantic region but its coastal distribution does not overlap with that of human and equine cases in New England. *Cq. perturbans* mosquitoes are commonly trapped in Connecticut (35,389 females collected in 2009) and are found near EEEV foci. Host-seeking females emerge as 1 generation that peaks in early July and then decrease sharply by mid-August when EEEV begins to amplify in Connecticut (*19*). EEEV has been isolated from *Cq*. *perturbans* mosquitoes throughout the eastern United States, including Connecticut in other years (*6**,**28**,**33**,**37*) (ArboNET). In the study by Nasci and Mitchell (*17*), 13 (65%) of 20 pools of *Cq. perturbans* mosquitoes contained high titers of EEEV (>3.0 log_10_ PFU/mL), which suggested a bridge role for this species. However, its low abundance in late August and September when EEE activity is greatest argues against its involvement as a primary epidemic vector in this region.

Our results obtained by qRT-PCR corresponded to those estimated by plaque titration, which provided a basis for interpreting C_t_ values estimated directly from mosquito pools. On the basis of the fitted line estimated by regression analysis in the Figure, a C_t_<29.8 corresponded to virus titers >3.0 log_10_ PFU/mL. We used this titer as a threshold for classifying low or high virus titers in mosquito pools for purposes of comparison and to discern whether a virus infection was established and replication occurred in the mosquito vector. Competent vectors must support virus replication during the extrinsic incubation period to be able to transmit virus. However, quantitative data for the minimum virus titer necessary for transmission are limited or absent for most virus–vector systems. Despite these uncertainties, investigations showed that mosquitoes transmitted virus when their body titers exceeded 4–5 logs of virus for EEEV (*23**–**25*) and western equine encephalitis virus (*38**,**39*). Virus transmission was usually associated with mosquitoes containing >5 logs of virus. However, some females with high virus titers failed to transmit virus in these studies. On the basis of these considerations, we believe that mosquito pools exhibiting low virus titers (<3.0 log10 PFU/mosquito pool) would be highly unlikely to contain mosquitoes capable of transmitting virus at the time of sampling, whereas detection of high virus titers does not necessarily predict that infected mosquitoes are capable of virus transmission. The strength of the data in our study is based on consistent detection of high virus titers from only 1 competent mosquito vector (*Cs*. *melanura*).

Most of the pools that showed EEEV in cell culture also showed positive results by qRT-PCR (C_t_ <37). However, our ability to reisolate EEEV from another 14 mosquito pools with either equivocal or negative results indicates that Vero cell culture is a highly sensitive assay for detection of EEEV. Mosquito pools containing infectious virus should be reconfirmed and quantified by another independent method; qRT-PCR is currently the most convenient method to accomplish this test.

Published data on virus titers from field-collected mosquitoes are currently limited to 1 study by Nasci and Mitchell (*17*), and their findings were consistent with our observations in *Cs*. *melanura*, *Ae*. *vexans*, and *Oc*. *canadensis* mosquitoes. Virus titer variation observed among mosquito species could reflect differences in vector competence. EEEV was shown to infect, replicate, and disseminate rapidly in *Cs*. *melanura* mosquitoes and was detected in the salivary glands 2–3 days after infection (*25*). Moreover, a larger proportion of *Cs. melanura* mosquitoes transmitted EEEV than *Aedes*, *Anopheles*, *Coquillettidia*, *Culex*, and *Ochlerotatus* mosquitoes in a direct laboratory comparison (*8*). Virus titers will also vary according to the duration of the extrinsic incubation period. One limitation of our study is that because we did not know the age structure of the mosquito population, we could not control this variable.

Low virus titers may have also been caused by contamination of infected mosquito fragments during sorting and testing procedures. Although we cannot preclude this possibility, we made concerted efforts to ensure accurate testing. Mosquitoes were sorted into mosquito pools on chill tables, and mosquito pools were processed for virus isolation in a separate facility according to standard practices. EEEV-positive pools were directly tested and quantified by using 2 independent tests (plaque titration and qRT-PCR). Our results were consistent with those of other studies (*1**,**28**–**35*), which showed that most EEEV isolations were from *Cs*. *melanura* mosquitoes and several other mosquito species.

Our findings highlight the need to consider virus titer when interpreting virus isolation or PCR detection data for field-collected mosquitoes. Although we isolated EEEV from several mosquito species, *Cs. melanura* was the only species that had consistently high titers of EEEV sufficient for transmission. This finding may help reconcile the paucity of symptomatic human and equine cases, despite frequent detection or isolation of virus from mammalophilic mosquitoes during episodes of virus amplification. These results should be verified in other regions, where involvement of other locally abundant mosquitoes is suspected during disease outbreaks. When information on virus titers in mosquitoes is considered, the number of candidate vectors may be reduced to a few key species that are capable of supporting virus transmission in nature.
